# The performance of anchovy purse seine in the North Coastal Java Sea, Indonesia

**DOI:** 10.1016/j.heliyon.2024.e33324

**Published:** 2024-06-28

**Authors:** Suparman Sasmita, Zarochman, Zainal Wassahua, Sri Suryo Sukoraharjo, Yopi Novita, Budhi Hascaryo Iskandar, Fis Purwangka, Ronny Irawan Wahyu, Mokhamad Dahri Iskandar, Iin Solihin, Rafi Ohorella, Nurdin Kasim, Muh Soghirun, Jacomina Tahapary, Pringgo Kusuma D.N. Y. Putra

**Affiliations:** a Research Center for Fishery, National Research, and Innovation Agency (BRIN), Jalan Raya Bogor Km. 47 Cibinong, 16912, Nanggewer Mekar, Bogor, West Java, Indonesia; b Department of Fisheries Resources Utilization, Faculty of Fisheries and Marine Sciences, Bogor Agricultural University, Jl. Agatis, Dramaga, Bogor, 16680, West Java, Indonesia; c Marine and Fisheries Polytechnic of Bone, Ministry of Marine and Fisheries Affair, Jl. Sungai Musi Km. 09, 92718, South Sulawesi, Indonesia; d Polytechnic State Fisheries Tual, Ministry of Education, Culture, Research, And Technology, Jl. Raya Langgur Sathean Km 6, 97611, Maluku, Indonesia; e Department of Fisheries, Faculty of Fisheries and Marine Science, Universitas Padjadjaran, Jl. Raya Bandung-Sumedang Km. 21, Jatinangor, 45363, West Java, Indonesia

**Keywords:** Anchovy, Buoyancy force, Purse seine, Sinking force, Sinking speed

## Abstract

Small pelagic fishes including anchovies in Indonesia are caught by using different types of fishing gears. However, the anchovy purse seine commonly used polyethylene (PE) as material webbing which is less effective in the North Coastal Java Sea. This study aims to describe the design and construction of the anchovy purse seine and the characteristics of this gear construction. The research was carried out in the North Coastal Java Sea of Indonesia between May and June 2021. The main data from the nine anchovy purse seine units were collected using purposive sampling. The sampling method was performed by identifying and measuring the components of the number of anchovy purse seiners. The ratio of the length of the head rope to the length of the ground rope is between 0.926 and 0.991, so the net is not exactly rectangular, but almost trapezium in shape. The anchovy purse seine was constructed using webbing polyamide (PA) with 8 mm–9 mm in mesh size, which is considered a small mesh size. It is assembled with some ropes, floats, and sinkers. The anchovy purse seine is characterized by the dominant buoyancy value, 48 % larger than the sinking force. The sinking speed is 0.15 m/s, and the sinking time is 164 s. The anchovy purse seine's design and construction are suitable for operating in the waters of North Coast of Java.

## Highlights

• The anchovy purse seine constructed using a polyamide (PA) small mesh size is effective for the anchovy target.
• The anchovy purse seine is characterized by the dominant buoyancy value, 48 % larger than the sinking force.
• The anchovy purse seine's design and construction are suitable for operating in the waters of North Coast of Java.

## 1 Introduction

The Fisheries Management Area (FMA) in the Republic of Indonesia has potential fish resources, which is FMA-712, such as demersal fishes, small pelagic fishes, large pelagic fishes, squids, shrimps, coral fishes, and crabs [1]. Generally, fish resources in Indonesian waters are dominated by two main fish groups: small pelagic fishes by 36 % and large pelagic fishes by 25 %. Indonesian fish resource in 2015 was estimated at 9.931 million tons/year, comprising 1.992 million tons/year (20 %) [2]. The fishermen caught small pelagic fish in FMA-712 as the targets. This area contains 364,663 tons/year as potential value of fish resources [1]. The anchovies are one of the main targets. Most are operated along Java's north coast, including in the FMA-712 [3,4]. The potential of small pelagic fish resources is important to manage because of their contribution to the national economy [[5], [6], [7]].

Various fishing gear operated actively and passively, depending on habitat types, often have size, species, and behavior of fishes [8,9]. The passive gears, such as lift nets, fix gillnets, lines, and traps [10]. The active gears include danish seines, trawls, purse seines, and push nets [8,11,12]. Purse seines operated in FMA-712 to catch pelagic fishes such as sardines and other small pelagic fishes [13]. There is a special purse seine to catch anchovy fishes. It uses small mesh-size nets by using a small boat in size. Most of them used fishing boats of less than 20 GT with the main engine as land used engine of more than 98,6 HP. They also used an auxiliary engine of less than 18 HP to operate for pulling net and lines of anchovy purse seine. The total crew numbers are 14–20 persons [14].

Anchovies can be classified as small pelagic fish that usually swim in schooling with other pelagic fish and spread more than one schooling. Anchovies swim around the coastal waters [[15], [16], [17]]. In general, anchovies are caught using bagan (the local name of lift net) with lights and payang [[18], [19], [20]]. The number of anchovies catches increases yearly in the Java Sea [21]. One of the fishing gears developed for catching anchovies is purse seine [3,22,23]. Various pelagic fish have been targeted by purse seine [24], such as *Decapterus macrosoma* (Deles kite), *Sardinella lemuru* (Lemuru), *Auxis rochei* (Lisong cob) [[25], [26], [27], [28], [29], [30], [31], [32], [33], [34]]. However, some Indonesian fishing areas use purse seine to catch anchovies [23,35].

A purse seine is a surrounding net operation, a rectangular or trapezium in shape, assembly with rings and purse line, operated by pulling the purse line to close the bottom of the net using one or two boats [36,37]. Purse seine net will be shaped like walls to encircle schooling pelagic fish in the surface layer [16,[38], [39], [40]]. Regarding the purse seine design, such as hanging ratio, length of head rope, and length of purse lines are considered important parameters [38,41].

The shape of the purse seine has dimensions and sizes, and each fisherman arranges different components and dimensions of the net [42]. Different sizes and numbers of components will give the net unequal characteristics. The construction of purse seine nets is distinguished by the technique of placing or a series of components of buoys, ropes, nets (webbing), sinkers, and rings [39,43,44].

This paper discusses the characteristics of purse seine on buoyancy force, sinking force, and sinking speed, which are critical to ensure the proper operation of purse seine and the capture of anchovies. The study aims to describe the design and construction of purse seine nets for catching anchovies and their characteristics in Pulolampes, District Brebes.

## 2 Materials and methods

Data collection was carried out in May–June 2021. The net type considered as the object of research is anchovy purse seine operated in Brebes Waters of the North Java Sea. The sampling method is a purposive sampling of the population of anchovy purse seiners. The number of samples used in this method was obtained from 10 % of the population of boats operating the anchovy purse seine. So, this sampling data can describe the performance of fishing boats related to fishing operations. Data were taken from 9 (nine) anchovy purse seiners (Table 1), among which had an average length overall (LOA) of 13.99 m, a width (Breadth/B) of 3.87 m, and a depth (Depth/D) of 1.22 m.

**Table 1:** The specifications of anchovy purse seiners (in meters).

No.	Names	LOA	B	D	L/B	L/D	Main Engine^a^ (HP)	Line Hauler Engine (HP)
1	KM. Guntur Mandiri Vega	15.30	4.47	0.98	3.42	15.61	98.60	24.00
2	KM. Fitri Mandiri	11.60	3.70	1.00	3.14	11.60	98.60	20.00
3	KM. Mutiara	14.00	3.80	1.40	3.68	10.00	118.32	20.00
4	KM. Makmur	14.00	3.60	1.30	3.89	10.77	118.32	23.00
5	KM. Sri Rahayu	14.00	3.60	1.30	3.89	10.77	147.90	22.00
6	KM. Mekar Sari	13.50	3.70	1.10	3.65	12.27	98.60	23.00
7	KM. Kontan Baru	14.00	4.20	1.40	3.33	10.00	118.32	20.00
8	KM. Sumber Makmur	14.55	3.85	1.10	3.78	13.23	118.32	20.00
9	KM. Tunggal Kontan	15.00	3.90	1.40	3.85	10.71	118.32	20.00

a used land engine.

This research will also describe the design and construction to calculate the anchovy purse seine's buoyancy force and sinking speed. The data in this research was obtained by measuring components in ropes, webbing, buoys, sinkers, and rings, including each component's dimensions and weight. The data requirements of components are diameter, length, weight, and material. The measurement weight of the rope in the air is obtained based on the values of length, diameter, and material [45], as presented in Eq. (1):

(1) $$W=L\times \frac{D^{2}}{4}\;\pi \times \rho_{b}\times k$$

*W*: Weight of objects in the air (kg)

*L*: Length of the rope (m)

D: Diameter of the rope (m)

π: 3.14

$\rho_{b}$: Weight of mass (kg/m^3^)

*k*: Coefficient volume PE = 0.63.

The weight of knotless webbing is measured directly in location. Some items for measurement include mesh sizes, bars, points, and the weight of webbing in the air.

To calculate the weight of the rope and webbing components in the water, the components of each part of the net must first be known as the type of material. The estimated weight in water is calculated based on the weight and density of each component [46], as presented in Eq. (2):

(2) $$P=A\;\left(1\;-\;\frac{D_{w}}{D_{m}}\right)$$

*P*: Weight of component materials in the water (kg)

*A*: Weight of the component material in the air in a dry state (kg)

*D*_*w*_: Water density (g/cc) (sea waters: *D*_*w*_ = 1.026)

*D*_*m*_: Density of component materials (g/cc)

The total buoyancy and sinking force are accumulated from each component's buoyancy force and sinking force. The calculation of buoyancy force depends on the density of the material [46], as presented in Eq. (3):

(3) $$B_{1}=W\;\left(\frac{\partial_{sw}}{\partial_{w}}-1\right)$$

*B*_1_: Buoyancy force of the components (kgf)*

*W*: Weight in the air of the components (kgf)

∂_*w*_: Density of components (gr/cm³)

∂_*sw*_: Density of seawater (gr/cm³)

*A component has a buoyancy force if the density of the component is smaller than the density of seawater (1.025 gr/cm^3^).

The sinking force of the components depends on the density of the material. By using the calculation formula [46], as presented in Eq. (4):

(4) $$S_{1}=W_{n}\;\left(1-\frac{\partial_{sw}}{\partial_{w}}\right)$$

*S*_1_: Sinking force of the components (kgf)

*W*_*n*_: Weight in the air of the components (kgf)

∂_*w*_: Density of components (gr/cm³)

∂_*sw*_: Density of seawater (gr/cm³)

The sinking speed of the anchovy purse seine depends on sinking force and sinking time. The average sinking speed of the net can be estimated using the following equation [47], as presented in Eq. (5):

(5) $$V=\frac{z_{m}}{T}$$

*V*: Sinking speed (m/min)

*z*_*m*_: Depth (m)

*T*: Sinking time (min)

The sinking time of the purse seine net is the time needed for all lead lines has reached maximum depth in accordingly all the stretched net area. It is calculated for one unit of fishing gear (anchovy purse seine). In order to hold the anchovy purse seine vertically, the sinking time value becomes a benchmark to determine the optimal time for fishing gear [47].

The limitation of this study was that it used knotless webbing in the waveless waters, so it was not needed to measure the sinking time of the gears. The sinking time can be expressed as the ratio of the net height to the average sinking speed.

## 3 Results

The net construction of the anchovy purse seine has a square shape consisting of webbing assembled with ropes, buoys, sinkers, and rings. Webbing has been arranged with 4.5 pieces in the vertical direction and 4 pieces in the horizontal direction. The bunt is a joining part of the anchovy purse seine on the horizontal side at the top of the webbing, and the last part is released from the ship during setting operations. The webbing material is PE and PA with a mesh size of 8 mm–9 mm knotless types of webbing. The types of net do not affect the increase in net weight but can increase sinking speed [[48], [49], [50]]. Webbing materials have different characteristics for example webbing made from PE has floating and light properties, but webbing made from PA has sinking and heavy properties. Webbing is joining with different types arranged horizontally and vertically. Purse seine construction has differences in the number of strips of webbing. The PA webbing comprises 4.5 joins horizontal strips and 4 joins vertical strips, and PE webbing comprises 4 joins horizontal strips, and 1 joins vertical strip (Table 2).

**Table 2:** Component (types, and materials) of anchovy purse seine.

Component (Types/Material)	Samples								
	1	2	3	4	5	6	7	8	9
Head Rope									
*Ø (mm)/PE*	10	10	8	8	8	10	8	8	6
*Lenght (m)*	337	218	337	345	325	335	325	337	325
Float rope									
*Ø (mm)/PE*	8	10	8	8	10	8	10	8	12
*Lenght (m)*	337	218	337	345	325	335	325	337	325
Ground rope									
*Ø (mm)/PE*	8	8	8	8	8	6	8	8	6
*Lenght (m)*	351	225	340	369	351	360	342	342	336
Sinker rope									
*Ø (mm)/PE*	10	10	6	6	8	6	8	6	6
*Length (m)*	351	225	340	369	351	360	342	342	336
Purse line									
*Ø (mm)/PE*	26	26	26	32	32	28	28	26	20
*Length (m)*	536	389	481	538	520	528	515	484	509
Webbings PA									
Numbers of strips									
- *Horizontal*	4.5	4.5	4.5	4.5	4.5	4.5	4.5	4.5	4.5
- *Vertical*	4	4	3	3	3	3	3	3	3
Numbers (piece)	18	18	13.5	13.5	13.5	13.5	13.5	13.5	13.5
Webbings PE									
Numbers of strips									
*- Horizontal*	4	4	4	4	4	4	4	4	4
*- Vertical*	1	1	1	1	1	1	1	1	1
Numbers (piece)	8	8	8	8	8	8	8	8	8
Floats TF-17/PVC (piece)	1264	888	1262	1183	1129	1119	1304	1262	1304
Floats Y-3/PVC (piece)	1686	960	1682	2394	504	–	2604	1682	2604
Local float/Rubber (piece)	422	242	421	320	875	1.119	–	421	–
Sinkers/Pb (piece)	1350	800	1890	1320	1256	1440	1140	1224	1200
Rings/Fe (piece)	100	97	100	110	100	111	105	100	90
Hanging Ratio	0.82	0.53	0.82	0.84	0.79	0.81	0.79	0.82	0.79

Note: (+) sinking; (−) floating; Ø: diameter.

The rope as a component in the purse seine is used as the head rope, float rope, ground rope, sinker rope, and purse line. According to Table 2, some of the ropes used were 8 mm in diameter, such as head, float, and ground ropes with various lengths. The rope should have strength and light properties, so it is possible for fishing operations. The floats assembled on the anchovy purse seine consist of 3 types, i.e., TF-17 using PVC material, Y-3 using PVC material and rubber. Some construction uses rubber as traditional floats. Fishermen assemble floats with an intermittent installation system between each type. A series of floats are tightly fixed without any distance from each other. Float type TF-17 with PVC material is installed with a total of 888–1304 pieces, each unit weight measuring 0.08 kg. The float type Y-3 with PVC material is assembled with 540–2604 pieces weighing 0.08 kg each.

The sinker consists of sinkers mounted along sinker lines and rings hanging on hanging ropes. The amount of sinker on purse seine varies and is at least 800 pieces with a unit weight between 0.20 and 0.30 kg. The ring on the purse seine has a role as a sinker to which the purse line is threaded the purse line. Metal rings attached to the lower edge of a purse seine to which the purse line is threaded [16]. The number of rings varies by at least 90 pieces with a unit weight of 0.51 kg (Table 2).

**Fig. 1:**
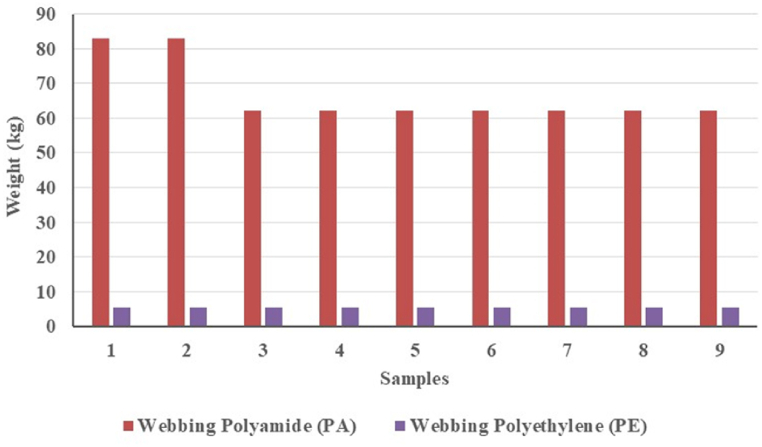
The estimated sinking force value of webbing on anchovy purse seine.

The sinking value depends on the total weight of the webbing material. The PA webbing for one piece weighs 46 kg in the air, of which it is estimated to have a sinking force value of 62.1 kgf - 82.8 kgf for one unit of anchovy purse seine. The PE webbing for one piece has a weight of 7 kg in the air, of which it is estimated to have a buoyancy force value of 5.6 kgf for one unit of anchovy purse seine (Table 2). The ratio of PA webbing to PE webbing on anchovy purse seine is 12 : 1. The webbing component of PA is more dominant than that of PE webbing (Fig. 1). The sinking force of the anchovy purse seine is higher, and the net will sink easily.

The buoyancy value was calculated based on a unit of anchovy purse seine for head rope ranges 0.44–1.27 kgf, float rope 0.81–1.76 kgf, ground rope 0.81–1.76 kgf, sinker rope 0.45–1.32 kgf, and purse line 7.68–20.71 kgf. Otherwise, the value of buoyancy force per unit length for head rope 0.0013–0.0038 kgf/m, float rope 0.0024–0.0054 kgf/m, ground rope 0.0014–0.0024 kgf/m, sinker rope 0.0014–0.0038 kgf/m, and purse line 0.015–0.0384 kgf/m.

**Fig. 2:**
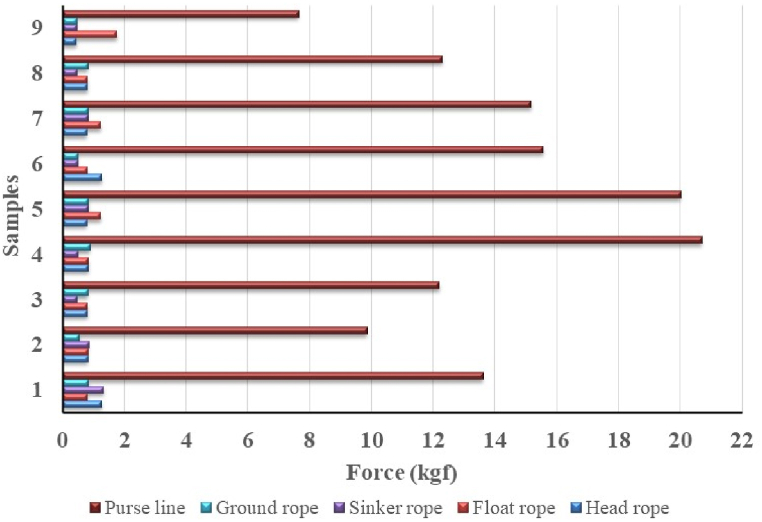
Calculated buoyancy force of rope of the anchovy purse seine.

**Fig. 3:**
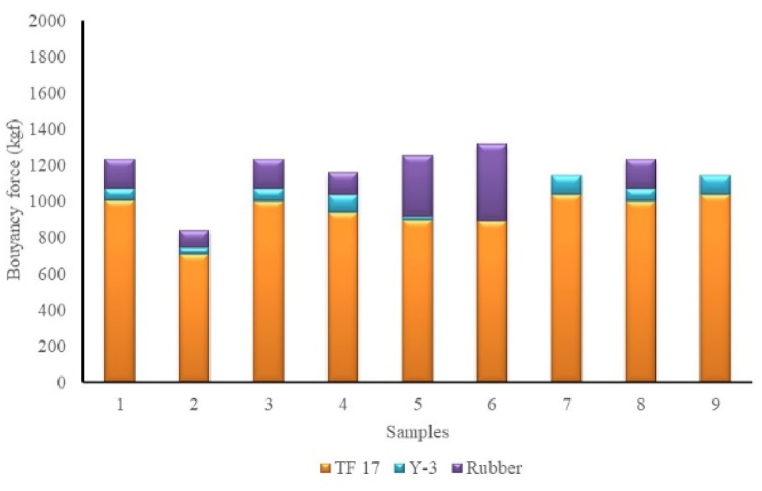
Total buoyancy value of float on anchovy purse seine.

The rope component on anchovy purse seine has a force value dominated by material PE and buoyancy properties. Based on the calculation, the buoyancy force value of the purse line is greater than other rope components (Fig. 2). The total buoyancy value of the float types of Y-3 ranging 20.16–104.16 kgf. Rubber-type floats assembled on anchovy purse seines reached 1119 pieces, the maximum number of floats (for the purse seine sample 6) with a weight size of 0.08 kg each. From this construction of rubber floats, it can be calculated that the buoyancy value is 426 kgf (Fig. 3). The floats on the anchovy purse seine are dominated by TF-17 type, rubber, and Y-3, respectively (Fig. 3). The number of TF-17 type floats tightly assembled without distance, in large quantities, will provide a high buoyancy force for the net. This condition gives less opportunity for anchovies to escape in the surface area.

**Fig. 4:**
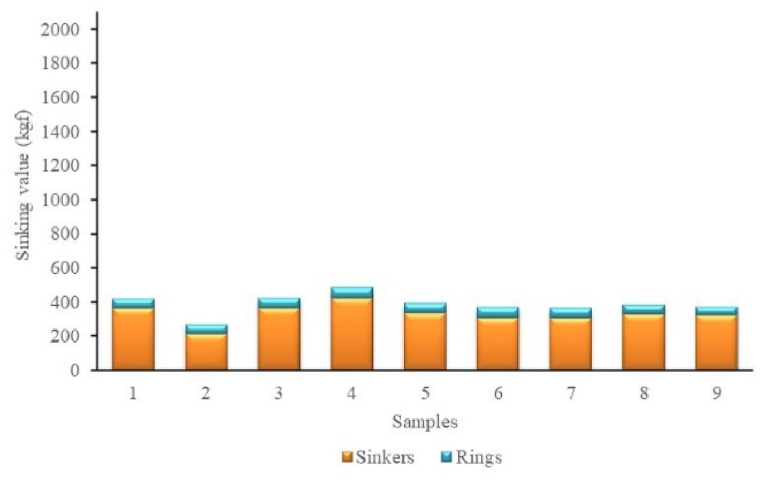
Total sinking value of sinkers and rings on anchovy purse seine.

The ring sinking force value on the purse seine ranges from 49.20 kgf to 60.69 kgf. The total sinking force of sinkers and rings along ground rope ranging 216.47–484.65 kgf (Fig. 4). Therefore, the operated anchovy purse seine has a higher buoyancy force. It is evidenced by the minimum value of the total buoyancy force of 858 kgf greater than the minimum value of the total sinking force of 269 kgf. The buoyancy force of each meter purse seine is more than 3.48 kgf/m, indicating that the head rope of the purse seine will float on the water's surface. The variation in the sinking force of sinkers and rings is illustrated in Fig. 4. The combined action of buoyancy force and sinking force in purse seine construction puts the net.

**Fig. 5:**
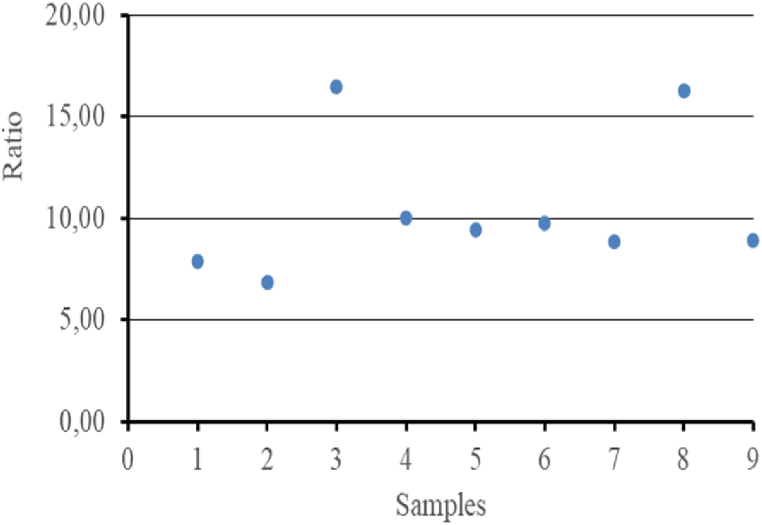
The ratio of buoyancy force to sinking force.

The difference between the maximum and minimum buoyancy force ratio is three times (Fig. 5). This difference indicates that the buoyancy force of the anchovy purse seine net construction is three times greater than the sinking force [33]. When the purse seine is operated, its float rope and head rope will float on the water surface and maintain the position of the webbing of the purse seine stretched down from the surface to the water layer required. The anchovy purse seine can sink speed depending on the height of the installed net and the value of the sinking force per meter on the sinker. Our research ignored the influence of environmental conditions because purse seine nets operate in waveless waters. If so, the value of the installed net height is 36.64 m with a per-meter sinking force value of 1.2 kgf, and then the sinking speed is obtained between 0.13 m/s to 0.17 m/s.

The length of anchovy purse seine depends on the boat size and float buoyancy required. So, a certain float type is installed with an appropriate arrangement to meet buoyancy needs. The boat size is required to adjust the boat's maneuvering ability when surrounding the net [51]. The length of the purse seine in the study was less than 15 times that of the boat. This condition occurred because the target of purse seine is anchovies. These fish have slow swimming speeds, so they do not require the length of the net.

**Fig. 6:**
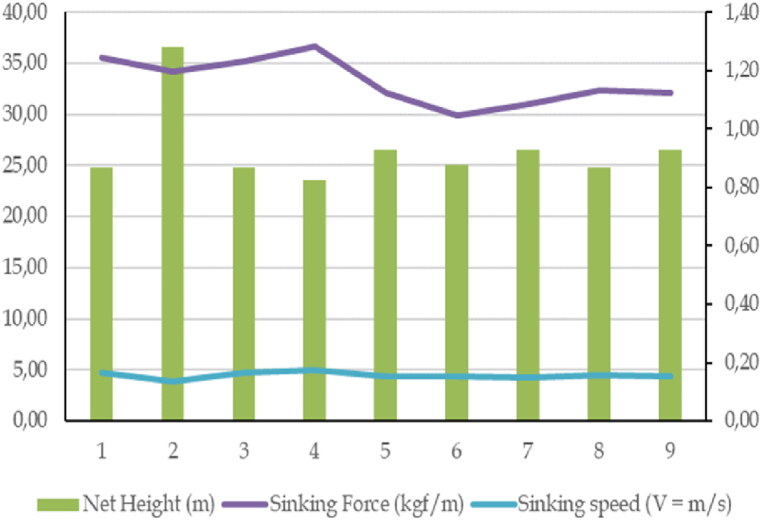
Sinking speed of anchovy purse seine.

The value of the sinking force of the anchovy purse seine varies, influenced by the sinking force and the height of the installed net. As the sinking force value increases, the sinking speed value increases (Fig. 6). Sinking time is obtained based on the ratio of sinking speed to the installed net height, where the value is obtained more than 135 s. Based on the estimation results in the calculation, the average sinking speed of the anchovy purse seine is 171.81 s. The value of the floating force of the anchovy purse seine is quite large compared to the sinking force, so the net will remain floating, and the anchovies escape in the surface water column will be smaller. The value of sinking speed and time will prevent the escape of anchovies already in the fishing area horizontally and vertically.

## 4 Discussion

Anchovys live in coastal waters and swim in groups [52]. The type of fish found in the *Engraulidae* family consists of 2 (two) subfamilies, *Coiliinae* and *Engraulinae*. The condition of the water environment strongly influences the abundance and distribution of many fish species [53,54]. The nature of swimming in schools of fish and the overall behavior of fish life will always be associated with the group [55,56].

Anchovy is one of the targets caught by fishermen with various tools such as lift net, surrounding net without purse line, and mini trawl. Fishermen modify their nets to increase catches and gain large profits by applying smaller mesh-size webbing. Anchovy schooling behavior spreads on the water's surface following zooplankton migration (5–20 m) [57]. Usually, anchovy purse seine in the Brebes Java Sea's north coast operates using a boat of less than 20 GT.

Based on the ship's dimensions with the highest L/B value and low L/D, the level of motion and layout on board is good for purse seine operations. Table 1 shows the highest L/B ratio value of 3.89 on purse seiners KM Makmur and KM Sri Rahayu and their shapes elongated. The ships with elongated shapes can move faster. Otherwise, when the ratio value of the L/D is 10.77, it is considered lower. It is more convincing for interior arrangement and longitudinal strength. In general, anchovy purse seiners used by fishermen in the northern coastal waters of the Java Sea have a high L/B and low enough L/D so that they can perform motion and layout to support the operation of anchovy purse seine properly.

**Fig. 7:**
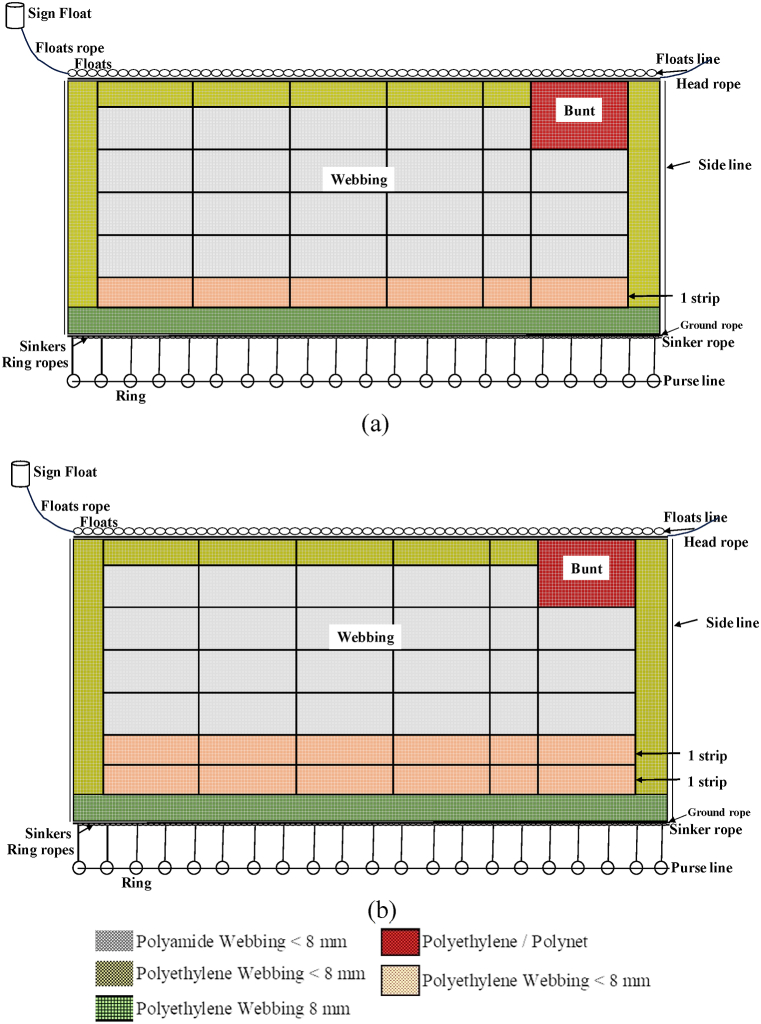
Webbing connector system of anchovy purse seine (a) one strip connector PE type; (b) two strips connector PE type.

The material of webbing determines the dimensions of the anchovy purse seine; most of the net series is made of PA, which has a sinking force with a small mesh size [4] so that the purse seine net can function as a wall to block the movement and escape of anchovy targets [40]. Another part of the net (webbing) made of PE adds a net area and reduces the net's weight while on board. There are 2 (two) type installations for the net at the bottom of the anchovy purse seine, namely 1 (one) strip of PE connection type as seen as Fig. 7 (a) and 2 (two) strips of PE connection type as seen as Fig. 7 (b). A sketch of the PA and PE net installations can be seen in Fig. 7.

The PA material webbing is widely used in purse seine with mesh sizes larger than 1 inch for various pelagic fish targets such as mackerel, scad fish, sardines, skipjack, and tuna [16,23,59,61,62]. In the Java and Madura sea waters, anchovies (Engraulidae) are caught using purse seine with a 0.5 inch mesh [14,16], surrounding net without purse line at the coded using PE with a 3/8 inch mesh [21] and the fixed bagan liftnet using PA material with small mesh net webbing [58]. The Anchovy purse seines using PA webbing material to make it easier to sink [33,42] to block fish targets from escaping from the catching area of the net. Therefore, the dimension net of anchovy purse seine is adjusted to the fishing vessel length and the water depth. The performance of anchovy purse seine is effective because of the design and as well as characteristics of the net material. The net mesh size, the diameter of twine, and the number of floats and sinkers can increase and decrease buoyancy at each part of the net. As a consequence, this can affect fishing operation behavior and performance of anchovy purse seine [60].

Based on the calculations, the rope component of the anchovy purse seine has little effect on buoyancy and sinking forces. The diameter and length of the rope vary greatly for both floating rope and head rope. The type of material, diameter, and length of the rope affects its weight in water [46]. The diameter of the rope varies between 8 and 12 mm, except for the purse line, which has a diameter of 26–32 mm. Some fishermen add a rope to the side and body of the net. The head rope determines the circumference of the purse seine when the net is operated in a circle, according to the trajectory and speed of the moving boat. When the rope is laid on the deck, the volume and weight on board are determined.

Floats and sinkers on anchovy purse seine influence buoyancy and sinking forces, strengthening the webbing stretch. The installation floats are closed to prevent fish from escaping above the net. Sinkers with a large sinking force are used to increase the sinking speed, preventing fish from escaping below the bottom of the net. The design form of the anchovy purse seine operated by fishermen in the northern coastal waters of Brebes Java Sea is a square resembling a trapezium shape.

The anchovy purse seine in the Northern Coastal Java Sea has more construction, materials, and operation advantages. The net construction of anchovy purse seine is dominated by PA net webbing combined with PE net webbing. The PA net webbing increases the sinking force value to block the anchovy from escaping from the net, causing the fishing operation to become faster and the bottom part of the net to not reach the sea bottom. Generally, the selectivity of fishing gear can be minimized bycatch from total catches [63]. The anchovy purse seine is operated during anchovy season to target anchovies as the dominant species specifically. This fishing gear is considered more selective for catching anchovies [3].

The anchovy purse seine operated in waveless conditions for further research is expected to determine the optimum value of oceanography for net performance. In addition, the selectivity of net mesh size is needed regarding various species of anchovies in the North Coastal Java Sea. Furthermore, zonation is necessary for managing the fishing ground and fishing time during the fishing operation.

## 5 Conclusions

The anchovy purse seine is operated by the net surrounding it to block anchovies schooling in shallow waters. The performance of this fishing gear design needs more sinking speed by using PA material. The number of PA materials in the four vertical strips could cause the net to sink faster, reducing the required sinking time. The net construction using 4.5 strips horizontally is suitable for the average length of fishing boats commonly used in local areas.

As a result, those vertical and horizontal constructions are suitable for anchovy purse seine regardless of the size of a fishing boat and the depth of water in that fishing ground. In the case of this study, it can be recommended that the performance of fishing gear be increased, as well as the suitable pulling speed of purse lines in anchovy purse seine operations to minimize escaping fish from catchable fishing areas.

## Data availability statement

Data will be made available on request.

## CRediT authorship contribution statement

**Suparman Sasmita:** Writing – original draft, Validation, Methodology, Investigation, Conceptualization. **Zarochman:** Writing – review & editing, Writing – original draft, Validation, Methodology, Investigation, Formal analysis, Conceptualization. **Zainal Wassahua:** Writing – review & editing, Writing – original draft, Validation, Methodology, Investigation, Formal analysis, Conceptualization. **Sri Suryo Sukoraharjo:** Writing – review & editing, Writing – original draft, Validation, Methodology, Investigation, Formal analysis, Conceptualization. **Yopi Novita:** Investigation, Formal analysis. **Budhi Hascaryo Iskandar:** Investigation, Formal analysis. **Fis Purwangka:** Writing – review & editing, Investigation. **Ronny Irawan Wahyu:** Writing – review & editing, Investigation. **Mokhamad Dahri Iskandar:** Visualization, Investigation. **Iin Solihin:** Visualization, Investigation. **Rafi Ohorella:** Visualization, Investigation. **Nurdin Kasim:** Visualization, Investigation. **Muh Soghirun:** Visualization, Investigation. **Jacomina Tahapary:** Visualization, Investigation. **Pringgo Kusuma D.N. Y. Putra:** Investigation, Formal analysis.

## Declaration of competing interest

The authors declare that they have no known competing financial interests or personal relationships that could have appeared to influence the work reported in this paper.
